# Myocardial impact and cardioprotective effects of apelin-13 and a c-terminal-modified analog during lps and clp experimental sepsis

**DOI:** 10.1186/2197-425X-3-S1-A436

**Published:** 2015-10-01

**Authors:** O Lesur

**Affiliations:** Université de Sherbrooke, ICU/ Medicine, Sherbrooke, Canada

## Introduction

Apelin-13 (APL-13) is a member of an endogenous peptide's family (APLs) with powerful inotropic and cardioprotective properties. APLs bind to the dedicated receptor APJ-R, a member of the G protein-coupled receptor superfamily, all being widely expressed in the cardiovascular system. We have already shown that APL-13 infusion, was protective against LPS-induced myocardial dysfunction and death vs. dobutamine [1]. Furthermore, we have shown that, C-terminal Phe(13) modification of APL-13 by unnatural amino acids can change ligand binding and APJ-R signaling [2].

## Objectives

Understanding the beneficial impact of APL-13 on LPS-induced myocardial injury vs. dobutamine, and assessing functional and biological effects of a new selected linear APL-13 analog with enhanced affinity, and their impact in the context of sepsis.

## Methods

Myocardial dysfunction was induced by intra-peritoneal injection of LPS (*E. Coli* 055:B5, 10 mg/kg) or Cecal Ligature and Puncture (CLP) in male Sprague-Dawley rats. Myocardial injury was biologically evaluated by analyzing of different cellular pathway of apoptosis and inflammation by Western blot. Myocardial function was assessed *ex-vivo* by Langendorff and *in vivo* by echocardiography by comparing APL-13 to Tyr(Obn). Tyr(OBn)(13) substitution led to a 60-fold increase in binding affinity vs. APL-13 [2].

## Results

LPS-challenged rats treated with APL-13 exhibited a clear reduction of both apoptosis (cleaved caspase-3, BAX/BCL-2 ratio) and inflammation (iNOS and MIF) markers, with significant alterations in the Akt/GSK3b/mTor and P38/Erk pathways underscoring the cardioprotective effect of APLs. Organic Langendorff assays confirmed cellular data [2] in that enhanced affinity confers to Tyr(Obn) analog a more effective and potent inotropic activity than APL-13, as shown by the increased left ventricular developed pressure (LVDP) (% baseline, 1pM : APL-13, 8 ± 13 vs. Tyr(Obn), 60 ± 15 ; p < 0.05), (30pM : APL-13, 124 ± 25 vs. Tyr(Obn), 372 ± 106 ; p < 0.05). APLs sensibility was increased in 8h CLP-challenged hearts, as it was in 24h LPS-challenged hearts [1], suggesting upregulation of the myocardial apelinergic pathway during polymicrobial sepsis. Indeed, CLP model of sepsis was characterized at 8h by a reduced cardiac output (Sham, 170 ± 3 vs. CLP, 99 ± 5 ml/min, p < 0.05) with an increased parietal thickness (Sham, 0.15 ± 0.002 vs. CLP, 0.23 ± 0.003 cm, p < 0.05) *in vivo*.

## Conclusions

APLs are new safer supporting drugs in sepsis. Chemical modifications can optimize the inotropic potency of APLs opening a novel field of therapeutic opportunities. Ongoing works are to evaluate the functional effect of these new analogs *in vivo*, with Pressure-Volume curve device, and to further test their comparative cardioprotective potential during experimental CLP sepsis.

## Grant Acknowledgment

nHSF, Merck
